# Sexual dimorphism of sonic apparatus and extreme intersexual variation of sounds in *Ophidion rochei* (Ophidiidae): first evidence of a tight relationship between morphology and sound characteristics in Ophidiidae

**DOI:** 10.1186/1742-9994-9-34

**Published:** 2012-12-06

**Authors:** Loïc Kéver, Kelly S Boyle, Branko Dragičević, Jakov Dulčić, Margarida Casadevall, Eric Parmentier

**Affiliations:** 1 Laboratoire de Morphologie Fonctionnelle et Evolutive, Institut de chimie, Bât. B6c, Université de Liège, B-4000, Liège, Belgium; 2 Institute of Oceanography and Fisheries, POB 500, 21000, Split, Croatia; 3 Grup de Biologia Animal, Facultat de Ciències, Universitat de Girona, Campus de Montilivi, 17071, Girona, Spain

**Keywords:** Ophidiidae, Sound production, Sexual dimorphism

## Abstract

**Background:**

Many Ophidiidae are active in dark environments and display complex sonic apparatus morphologies. However, sound recordings are scarce and little is known about acoustic communication in this family. This paper focuses on *Ophidion rochei* which is known to display an important sexual dimorphism in swimbladder and anterior skeleton. The aims of this study were to compare the sound producing morphology, and the resulting sounds in juveniles, females and males of *O*. *rochei*.

**Results:**

Males, females, and juveniles possessed different morphotypes. Females and juveniles contrasted with males because they possessed dramatic differences in morphology of their sonic muscles, swimbladder, supraoccipital crest, and first vertebrae and associated ribs. Further, they lacked the ‘rocker bone’ typically found in males. Sounds from each morphotype were highly divergent. Males generally produced non harmonic, multiple-pulsed sounds that lasted for several seconds (3.5 ± 1.3 s) with a pulse period of *ca*. 100 ms. Juvenile and female sounds were recorded for the first time in ophidiids. Female sounds were harmonic, had shorter pulse period (±3.7 ms), and never exceeded a few dozen milliseconds (18 ± 11 ms). Moreover, unlike male sounds, female sounds did not have alternating long and short pulse periods. Juvenile sounds were weaker but appear to be similar to female sounds.

**Conclusions:**

Although it is not possible to distinguish externally male from female in *O*. *rochei*, they show a sonic apparatus and sounds that are dramatically different. This difference is likely due to their nocturnal habits that may have favored the evolution of internal secondary sexual characters that help to distinguish males from females and that could facilitate mate choice by females. Moreover, the comparison of different morphotypes in this study shows that these morphological differences result from a peramorphosis that takes place during the development of the gonads.

## Background

Acoustic communication in the Teleostei has been studied extensively over the last six decades [1-5]. By 1981, Myrberg [6] had documented sound production in more than 30 families, including: Batrachoididae, Carangidae, Scianidae, Holocentridae, and Serranidae. More recently, sounds were recorded in additional taxa, such as Carapidae [7], Ophidiidae [8,9], Chaetodontidae [10,11], Oplegnathidae [12], and Sebastidae [13]. Communication sounds are now estimated to occur in as many as 109 teleost families [14]. Of note, these discoveries of fish acoustic communication have provided new examples of an increasing diversity of fish sound-producing mechanisms [15,16] that contrast with the relatively conserved mechanisms of sound emission in other vertebrate classes [17].

In addition to descriptions and identification of fish calls, several studies have examined how abiotic and biotic factors influence sound characteristics. Temperature affects sound production in many species, increasing the contraction rate of sound-producing muscles [18,19]. In *Cynoscion regalis*, higher temperatures increase pulse rate, call intensity, and the dominant frequency of the sound [20]. Similar effects on sound were also demonstrated in *Opsanus tau*[21] and *Ophidion marginatum*[22]. In the catfish *Platydoras armatulus*, the dominant frequency and, to a lesser extent, pulse period of drumming sounds were affected in a comparable manner [23]. Muscle features can influence sound characteristics as well. Differences in muscle length can cause changes in sound characteristics between juveniles and adults because of a body size-related scaling effect [24,25]. The twitch contraction time was found to rise with increasing body size in a salamander [26], lizard [24], and fish [25]. Besides size effects, the sound-generating mechanism of many fishes, especially the sonic muscles, is also sexually dimorphic [15]. In sciaenid species such as *Micropogonias undulatus*, the sonic muscles and associated swimbladder are larger in males [27,28]. In other species of the family, however, drumming muscles are completely lacking in females [27,28]. In three sciaenid species investigated by Hill, sonic muscles form before or during puberty depending on the species [27]. In the Batrachoididae, *Opsanus tau*[29] and *Porichthys notatus*[30,31], sexual dimorphism of sonic muscles is quite pronounced. In both species, sonic muscles are present in small juveniles [31,32] and differences observed in adult morphotypes are caused by differences in fiber growth rates and proliferation [29,31]: sonic muscles become bigger in males [29,31]. In addition, some fish species display a hypertrophy of sonic muscles during breeding season [33,34]. In weakfish, this hypertrophy results in the emission of sounds with higher intensities, lower frequencies, and longer pulse durations [20].

According to Nielsen *et al*. [35], Ophidiiformes comprises four families: Ophidiidae, Carapidae, Bythitidae, and Aphyonidae. Ophidiidae [36,37], Carapidae [2] and Bythitidae [38,39] are hypothesized to be soniferous fish based mainly on their morphology. Sounds were, however, recorded in five carapid species [7,40,41] and in two species of Ophidiidae [8,9,22].

Ophidiiformes present an extraordinary variety of highly specialized structures associated with sound production [2,9,37,42-45]. Moreover, the sonic mechanisms of Ophidiidae are characterized by pronounced sexual dimorphisms [36,37,42,45,46]. They are generally composed of modified thoracic vertebrae, one to three pairs of sonic muscles, and a highly modified swimbladder. In some species, the anterior part of the swimbladder forms a so-called ‘rocker bone’ [42,43,46,47] on which sonic muscles insert [9,16,42,43]. This structure is likely involved directly with sound production, as its movement deforms the swimbladder wall [2,9,16]. It is present in adult males of some *Ophidion* but not in females or in juveniles [42,46]. A rocker bone was also reported in carapids of the genus *Onuxodon*[2,48], but its presence in both sexes was not investigated. Sexual dimorphisms of sonic mechanism have been documented in many Ophidiidae, but sounds have only been recorded from males of two species. Thus, the associated influence of sex specific morphology on sound emission has not been determined.

The present work focuses on *Ophidion rochei*, an endemic species living in Mediterranean and Black Seas [49]. This species inhabits coastal shallow waters [35] but little is known about its biology because it is nocturnal and hides during daylight hours in the sand [50-52].

Because of its nocturnal habits, *Ophidion rochei* may provide insight on the evolution of acoustic communication in environments where available light limits visual communication. Moreover, most ophidiiform species inhabit deep seas [35] and thus may experience similar evolutionary selection pressures associated with living in a dark environment. Consequently, an understanding of the biology of sound production in *O*. *rochei* may provide important framework for hypotheses in future studies on acoustic communication in deep sea species.

The aims of this paper are: 1) to determine the different sonic apparatus morphotypes in this species 2) to obtain and describe sounds for each morphotype, and 3) to investigate the relationship between morphology, sound characteristics, and ecological niche in *O*. *rochei*.

## Results

### Gross morphology of the sonic apparatus

The sonic apparatus of male *Ophidion rochei* was described by Parmentier *et al*. [9]. The following description focuses on the differences between males, females, and juveniles. The terms given to the female and juvenile structures are based on their homology with male structures.

#### Anterior skeleton

**Figure 1:**
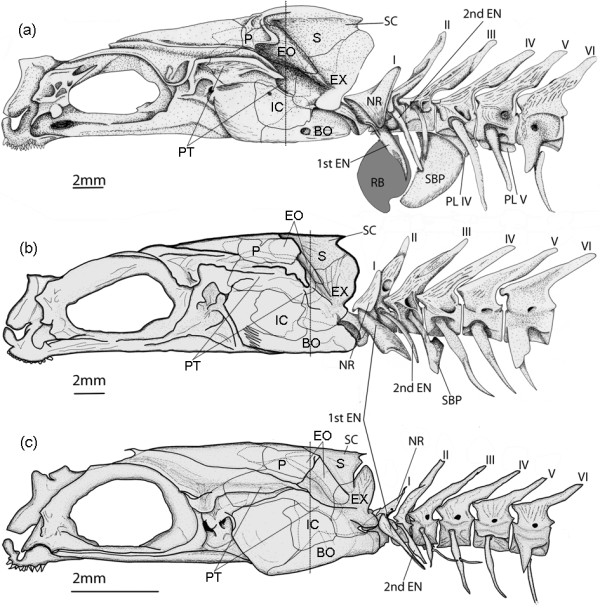
**Morphology of hard tissues in sonic apparatus of *Ophidion rochei.*** Left lateral view of the sound-production apparatus of (**a**) male (modified from [9]), (**b**) female and (**c**) juvenile *Ophidion rochei.* The skull, first six vertebrae and their associated ribs are shown*.* BO: basioccipital bone, EN: epineural, EO: epiotic bone, EX: exoccipital bone, IC: intercalarium bone, NR: neural rocker, P: parietal bone, PL: plate, PT: pterotic bone, RB: rocker bone, SBP: swimbladder plate, SC: supraoccipital crest, S: supraoccipital bone.

In *Ophidion rochei*, skeletal structures involved in sound production are located at the level of the head and the first five vertebrae (Figure 1).

The main differences between males and females are the following:

1. The occipital region is proportionally less developed in females than in males: the supraoccipital crest is thinner and lower, exoccipital and epiotic are smaller (Figure 1).

2. The sexual dimorphism of bony structures also appears at the level of the first vertebrae and associated ribs.

a) In all specimens, the first neural arch (or neural rocker) does not have a neural spine (Figure 1), and is horseshoe shaped. Both branches of this structure articulate with the vertebral body so that it is capable of pivoting in the antero-posterior plane. It has two large transverse plates that are firmly attached by connective fibers to the first epineural (called the wing-like process). In males, the rostral face and lateral sides of the neural rocker are higher and wider than in females. In both sexes, articulations of the first epineural on the vertebral body are hidden by the lateral parts of the neural rocker. The first epineurals point in the same direction in both sexes but their proximal parts are wider in females (Figure 1).

b) No difference was observed at the level of the second vertebra and associated epineurals (Figure 1). However, the distal tip of the 2nd epineural reaches the swimbladder plate only in males.

c) In males, the third epineural shows an osseous stem that articulates with the vertebral body proximally and a distal part that expands into a widespread convex bony plate (the swimbladder plate) associated with the tissues of the swimbladder. In females, the plate has a rectangular shape and is much smaller than in males. In males, the ventral plates of vertebrae 4 and 5 form a functional extension of the swimbladder plate. These ventral plates are absent in females (Figure 1).

d) Epineurals 4 and 5 are quite similar in both sexes (Figure 1).

The skeleton of the juvenile sonic apparatus is roughly similar to the females (Figure 1). The distal swimbladder plate at the level of the third epineural, however, is not yet developed at this stage. The contact between the third epineural and the swimbladder is restricted to the distal tip of the epineural. Though the neural rocker is present, its anterior and lateral parts are very thin and do not cover the proximal part of wing-like process (Figure 1).

#### Swimbladder

**Figure 2:**
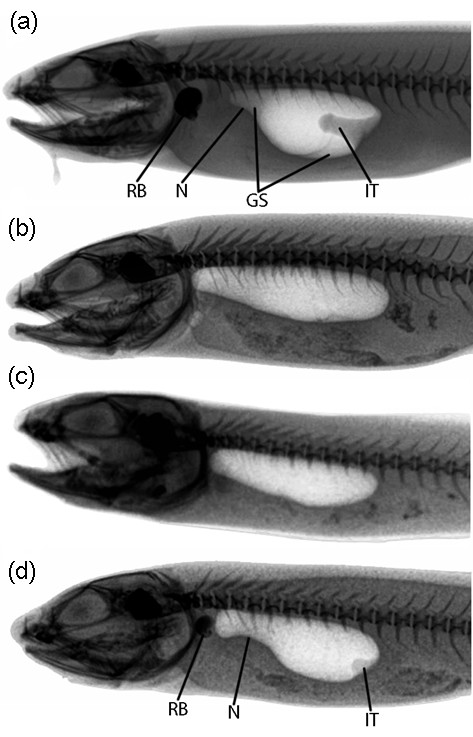
**Morphotypes observed on X-ray photographs of *Ophidion rochei. ***Photographs highlight the swimbladder and mineralized structures (as the skeleton, rocker bone, otoliths). Negatives of photographs are displayed to facilitate observation. (**a**-**c**) Typical morphotype of (**a**) male, (**b**) female, and (**c**) juvenile *Ophidion rochei*. (**d**) Intermediary morphotype hypothesized to be a young male *O. rochei*. Modifications of the swimbladder are evident in photographs of males: the rocker bone (RB), the internal tube (IT), the neck (N), and the gelatinous substance (GS). Radiographed specimens measured (**a**) 206 mm, (**b**) 197 mm, (**c**) 116 mm, and (**d**) 153 mm in TL.

**Figure 3:**
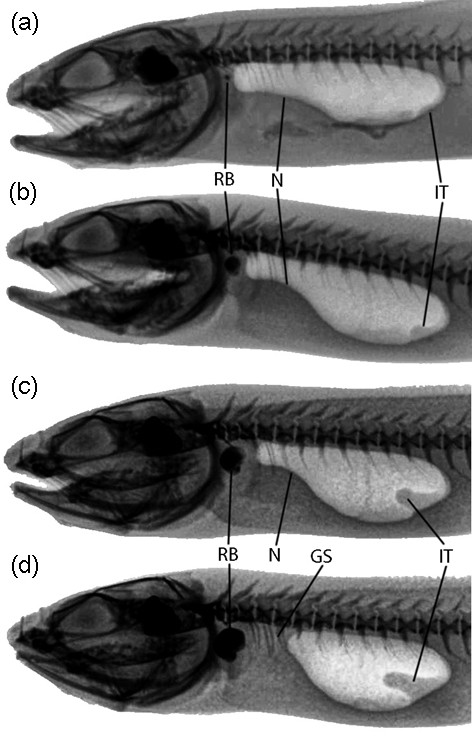
**Ontogenetic swimbladder modifications of a young male *Ophidion rochei.*** X-ray photographs from a growing male over the period of October 2010 to September 2011. Negatives of photographs are displayed to facilitate observation. The development of the rocker bone (RB), the internal tube (IT), and the neck (N) are indicated on the photographs. The gelatinous substance (GS) is clearly apparent in last picture (**d**). The specimen measured (**a**) 162 mm TL in October 2010, (**b**) 166 mm in March 2011, (**c**) 168 mm in May 2011, and (**d**) 170 mm in September 2011.

Male and female morphotypes could be distinguished clearly by X-ray photographs (Figures 2 and 3). Radiographs also allowed for an investigation of sonic apparatus development from juvenile to adult stages.

The swimbladder of males can be divided into two regions: anterior and posterior. The anterior region forms a neck and possesses a rocker bone at the rostral end. The posterior region is large and typically bears an internal tube (Figure 3) at its caudal end (see also, [9,46]). Furthermore, the anterior and posterior chambers of biggest males are filled with a gelatinous substance visible on radiographs (Figure 2 and 3). Female and juvenile swimbladders are ovoid-shaped, do not possess an anterior neck, internal structure, or rocker bone (Figure 2).

X-ray photographs taken in August 2010 on all specimens (juveniles and adults) from Croatia did not display typical male swimbladder structures. However, X-rays performed on the same fish in May 2011 displayed sexually dimorphic morphologies: 6 fish, 13.3 to 20.5 cm TL (total length), with male characteristics and 5 fish, 11.4 to 21.1 cm TL, with female morphology. Follow up observation of a specimen growing over a complete year revealed the development of the rocker bone, the neck, the internal tube, and the appearance of the gelatinous substance (Figure 3).

**Figure 4:**
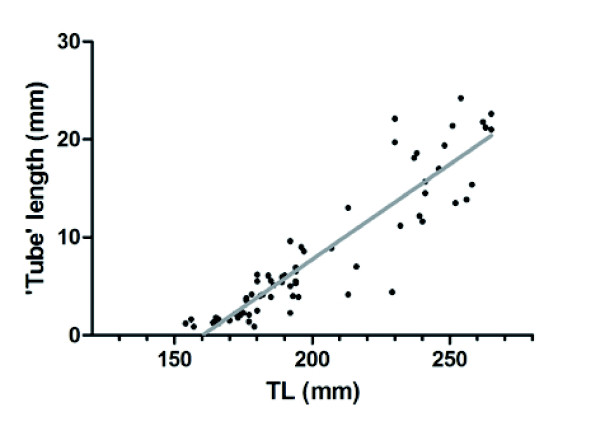
**Relationship between swimbladder internal tube length and body size in male *Ophidion rochei.*** There was a high correlation (r^2^ = 0.83 (N = 68), p < 0.0001) between the internal tube length and the total length of the fish. Black dots: observations for the 68 males *Ophidion rochei* from Costa Brava. Grey line: regression line (y = 0.1944x – 31.14) calculated for the 68 male swimbladders.

The study of swimbladders from fish caught along Costa Brava confirmed that adult females (153 to 265 mm TL, N = 20) are devoid of the internal tube as shown in Casadevall [46]. In males, the internal tube length is highly correlated (r^2^ = 0.83 (N = 68), p < 0.0001) with fish total length (Figure 4). The x axis intercept from the regression indicated an estimated total length of 160 mm (Figure 4) for the size at which the internal tube is expected to begin development.

Compilation of these data confirmed that male sonic apparatus characteristics are absent in juveniles and developed during maturation.

#### Muscles and ligaments

**Figure 5:**
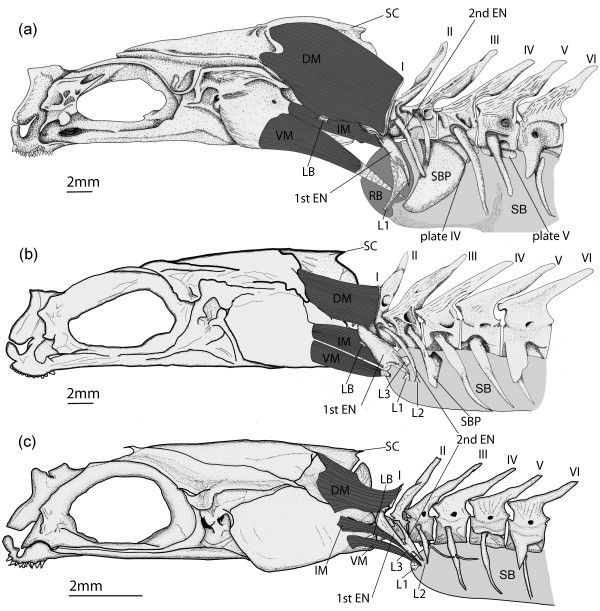
**Morphology of hard and soft tissues in sonic apparatus of *Ophidion rochei.*** Left lateral view of the sonic apparatus of (**a**) male, (**b**) female and (**c**) juvenile *Ophidion rochei.* The skull, the first modified vertebrae and ribs, and the 3 pairs of sonic muscles are shown*.* DM: dorsal sonic muscle, EN: epineural, IM: intermediate sonic muscle, L1-3: ligament 1–3, LB: ligament of Baudelot, RB: rocker bone, SB: swimbladder, SBP: swimbladder plate, SC: supraoccipital crest, VM: ventral sonic muscle.

Three pairs of sonic muscles were observed in each group (Figure 5). Precise descriptions of the male muscular system are given in Parmentier *et al*. [9] and are briefly provided here.

In males and females, the dorsal sonic muscle (DM) originates on the neurocranium and inserts on the neural rocker of the first vertebra. DM, however, was larger in males than in females (Figure 5). Furthermore, the supraoccipital crest and the anterior side of the horseshoe-shaped neural rocker are more developed in males (Figure 1). Ventral sonic muscles (VM) of males originate on a wider area of the skull and extend more rostrally compared to VM of females (Figure 5). In both sexes, they originate principally on the basioccipital and to a lesser extent on the intercalarium and the exoccipital. The posterior insertions of the VM are tendinous in both sexes. In males, VM insert on the posteroventral areas of rocker bone. In females, they insert on anterior parts of the swimbladder (Figure 5). Regarding intermediate sonic muscles (IM), they extend from the exoccipital bone to the wing-like processes in females, whereas they originate both on exoccipital and intercalarium bones in males (Figures 1 and 5). In addition, IM are fixed on the anterior proximal part of the wing-like processes in both sexes but the insertion areas are longer in females (Figure 5). Distal extremities of these epineurals are connected by a ligament to the rocker bone or to the swimbladder wall in males and females, respectively (Figure 5). Additional ligaments are present in females: ligament 2 connects the second epineural to the swimbladder wall and ligament 3 connects the 1st epineural to the 2nd epineural (Figure 5).

Juvenile morphology is similar to females (Figure 5). However, sonic muscles are much thinner in juveniles than in females (Figure 5).

### Sound recordings

#### Male sounds

**Figure 6:**
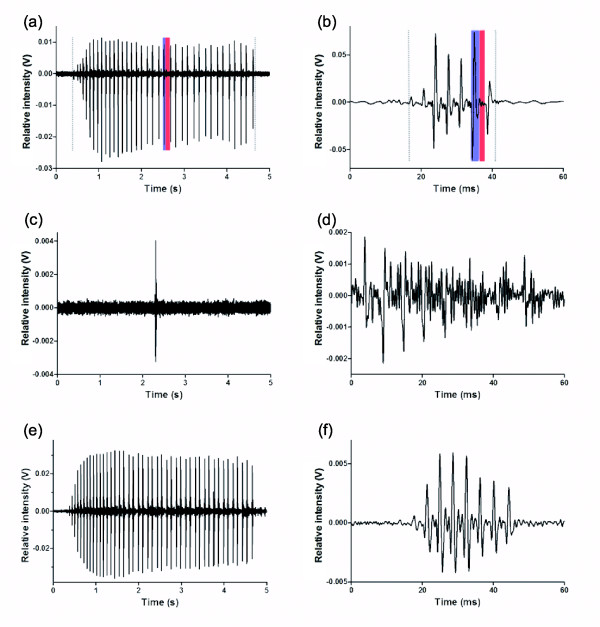
**Waveforms of male, female, and juvenile sounds in *Ophidion rochei*.** (**a**) 37 pulse sound of male in captivity, (**b**) 7 pulse female sound in captivity, (**c**) single pulse sound of male in captivity, (**d**) juvenile 8 pulse sound in captivity, (**e**) 44 pulse sound of male in the field, (**f**) 8 pulse female sound in the field. Grey dotted lines in (**a**) and (**b**) delimit the sound or call duration. Blue zones highlight a pulse (**a**) in a male sound and (**b**) in a female sound. Red zones highlight an inter-pulse (**a**) in a male and (**b**) in a female sound.

Multiple-pulsed sounds (Figure 6) were isolated from tank and field recordings. In both cases, the mean call duration was between 3.5 s and 4 s (Table 1), and was significantly (p < 0.05) correlated to pulse number (r^2^ = 0.86 and r^2^ = 0.59, respectively). The pulse period duration increased progressively in the first part of the sound before alternating between long and short durations. The alternation started at the 15th ± 3 (N = 26) pulse in captivity and 14th ± 2 (N = 20) in the field. In tank recordings, the number of pulses in a call fluctuated between 3 and 55 (median: 31 ± 10.3, N = 29) and field recordings varied from 31 to 53 (median: 40.5 ± 6.3, N = 20). Call duration and alternation start were not significantly different (p > 0.05) between the field and the tank sounds but pulse number, pulse period, and pulse duration were (Table 2). However, means and standard deviations of all temporal variables were similar in both recording situations (Table 1).

**Table 1:** **Principal characteristics of multiple-pulsed sounds of male *Ophidion rochei***

		**N**	**Mean**	**SD**
**Pulse number**	Capt.	29	**32**	10
	Field	20	**41**	6
**Alternation start**	Capt.	26	**15**	3
	Field	20	**14**	2
**Call duration (ms)**	Capt.	29	**3518**	1277
	Field	20	**3888**	762
**Short pulse period (ms)**	Capt.	261	**112**	10
	Field	278	**97**	11
**Long pulse period (ms)**	Capt.	254	**143**	18
	Field	276	**118**	10
**Pulse duration (ms)**	Capt.	921	**16**	13
	Field	821	**20**	8

**Table 2:** **Statistical comparisons between field and laboratory recordings for male multiple-pulsed sounds from *O. rochei***

**Variables**	**Methods**	**Results**
**Pulse number**	*t*-test	N = 49, t(47) = 3.57, p < 0.05
**Alternation start**	*t*-test	N = 46, t(44) = −1.4, p > 0.05
**Call duration**	*t*-test	N = 49, t(47) = −1.15, p > 0.05
**Short pulse period**	M-W *U* test	N = 564, z = −14.98, p < 0.05
**Long pulse period**	M-W *U* test	N = 555, z = −15.19, p < 0.05
**Pulse duration**	M-W *U* test	N = 1742, z = −16.74, p < 0.05
**1**^**st**^**main frequency**	M-W *U* test	N = 46, z = −2.52, p < 0.05
**2**^**nd**^**main frequency**	M-W *U* test	N = 47, z = −2.67, p < 0.05

*T*-test: the *t*-test of Student. M-W *U* test: *U* test of Mann–Whitney. Significance level is determined at p < 0.05.

**Figure 7:**
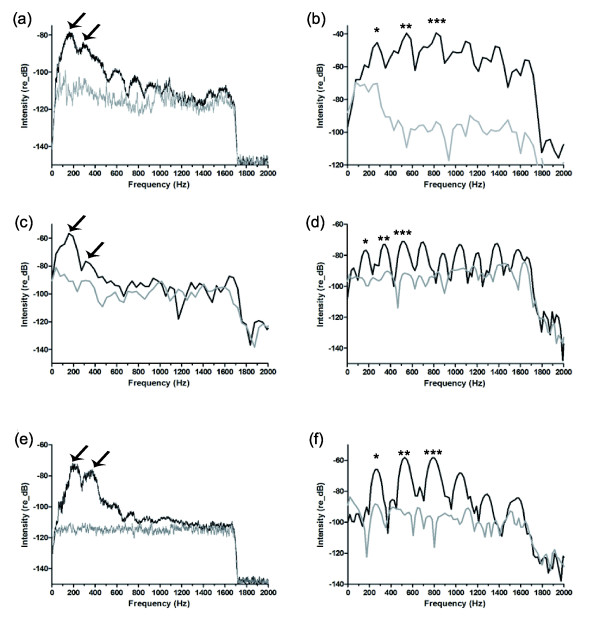
**Power spectra of male, female, and juvenile sounds in *Ophidion rochei.*** Grey lines: logarithmic power spectra of background noise. Black lines: logarithmic power spectra of (**a**) a multiple-pulsed sound of male in captivity (smoothed: average over 41 points), (**b**) a female sound in captivity, (**c**) a single pulse sound of male in captivity, (**d**) a juvenile sound in captivity, (**e**) a multiple-pulsed sound of male in the field (smoothed: average over 41 points), and (**f**) a female sound in the field. In power spectra that suggested a harmonic pattern (*) the fundamental, (**) the 1st harmonic, and (***) the 2nd harmonic were marked. In other power spectra the two arrows indicate the two main frequencies.

**Figure 8:**
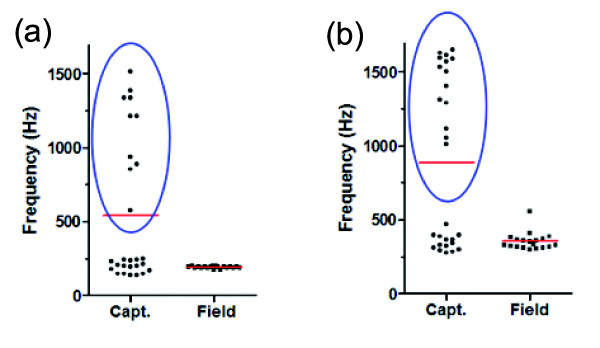
**Distribution of male *Ophidion rochei *sounds based on their spectral data.** These graphs represent the distribution of male (**a**) 1st and (**b**) 2nd main frequencies in captivity (capt.) and in the field. Red lines: illustrated mean values. Blue ellipses: encircled observations that displayed unusually high frequencies.

Power spectra of male multiple-pulsed sounds revealed no harmonic patterns (Figure 7) as evidenced by sound energy that was generally concentrated in two peaks with no consistent mathematical relationship between them. In captivity, the means were 541 ± 493 Hz (N = 27) and 884 ± 570 Hz (N = 28). It was 191 ± 7 Hz (N = 19) and 355 ± 59 Hz (N = 19) in the field. Despite the higher frequencies present in some sounds recorded in captivity, shapes of power spectra from field and tank sounds are quite similar (Figure 7). In captivity, peaks present at high frequencies could be due to tank resonance. The calculated resonance for glass aquaria of the size used in this study is greater than 1.7 kHz, however, our aquaria were made of plastic. Thus it is possible that the difference in materials may contribute to high frequency resonance. Some sounds (blue ellipses in Figure 8) recorded in aquaria possessed high frequency peaks (>500 Hz). The remaining sounds, however, possessed a similar distribution of frequency peaks as sounds in the field: 1st main frequency 195 ± 40 Hz (N = 17) and 2nd main frequency 347 ± 55 Hz (N = 14).

**Figure 9:**
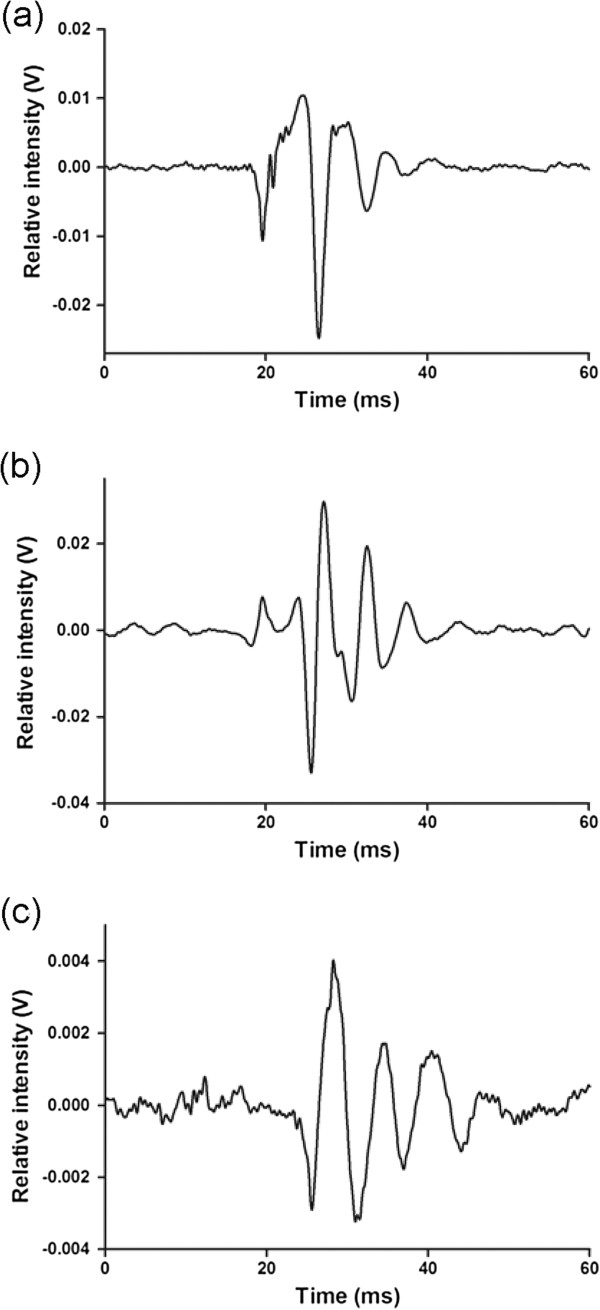
**Waveforms of *Ophidion rochei *individual male pulses.** Detail of a pulse from (**a**) a multiple-pulsed sound of male *Ophidion rochei* recorded in captivity, (**b**) a multiple-pulsed sound of male *Ophidion rochei* recorded in the field, and (**c**) a single pulse sound of male *Ophidion rochei* recorded in captivity.

Single pulse sounds (Figure 6 and 9) were recorded in captivity but not in the field. Their waveform was similar to the waveform of pulses isolated from multiple-pulsed sounds (Figure 9). In addition, the pulse duration was 12.4 ± 6.3 ms (N = 17) in single pulse sounds while it was 16 ± 13 ms (N = 921) for multiple-pulsed sounds in captivity. Spectral data showed high variability for 2nd main frequency (1068 ± 417 Hz, N = 17). However, the 1st main frequency (180 ± 48 Hz, N = 17) was less variable and closer to the 1st main frequency in the field (191 ± 7 Hz, N = 19). The power spectrum of a single pulse sound was shown in Figure 7.

#### Female sounds

Sounds produced by 4 adult females were first identified in tanks (Figure 6). They were produced spontaneously during nighttime but associated fish behaviors remain unknown. Temporal and spectral data of these sounds (Table 3) were used to identify female sounds in field recordings (Figure 6).

**Table 3:** **Principal characteristics of female sounds from *Ophidion rochei***

		**N**	**Mean**	**SD**
**Pulse number**	Capt.	56	**5**	3
	Field	20	**6**	3
**Call duration (ms)**	Capt.	56	**18**	11
	Field	20	**20**	10
**Pulse period (ms)**	Capt.	249	**4,0**	1,2
	Field	105	**3.7**	0.7
**Pulse dur. (ms)**	Capt.	310	**0.7**	0.2
	Field	121	**0.8**	0.4
**Fundamental (Hz)**	Capt.	50	**246**	28
	Field	18	**249**	23
**1**^**st**^**harmonic (Hz)**	Capt.	57	**506**	83
	Field	19	**494**	45
**2**^**nd**^**harmonic (Hz)**	Capt.	58	**779**	114
	Field	19	**763**	81

Females emitted shorter calls (18 ± 11 ms, N = 56 in captivity and 20 ± 10 ms, N = 20 in the field) than males. Mean call duration of female sounds was similar to the mean duration of individual pulses from male calls. However, female sounds were described as multiple-pulsed sounds because they were composed of a highly variable number of repeated units that had a relatively stereotyped waveform (Figure 6). This was not observed in male pulses (Figure 9). The number of pulses in female sounds varied from 2 to 12 in captivity and from 3 to 13 in the field. In captivity, the pulse period was 4 ± 1.2 ms (N = 249) and the pulse duration was 0.7 ± 0.2 ms (N = 310). These variables were similar in the field: 3.7 ± 0.7 ms (N = 105) and 0.8 ± 0.4 ms (N = 121), respectively. Spectral characteristics of female sounds strongly suggested that they were harmonic (Figure 7). The fundamental frequency was 246 ± 28 Hz (N = 50) in captivity and 249 ± 23 Hz (N = 18) in the field. There is a direct relationship between the fundamental frequency and the pulse period. Indeed pulse period is close to the inverse of fundamental frequency (Table 4). Moreover, in each case, these sounds presented at least a first and a second harmonic (Table 3 and Figure 7).

**Table 4:** **Measured and calculated pulse periods of female sounds from *Ophidion rochei***

		**Captivity**	**Field**
**Pulse period (ms)**	Measured (automatically during sound analysis)	4.0	3.7
	Calculated (1/fundamental frequency)	4.1	4.0

Call duration, pulse number, pulse period, fundamental frequency, and 1st harmonic did not differ significantly between calls from the field and captivity but pulse duration and 2nd harmonic did (Table 5). These results tend to confirm the identification of female sounds on field recordings. The observed differences in pulse duration should be considered with caution because of the extremely short pulse duration in calls from both environments. Further, the differences between pulse duration are about 10^-4^ second (Table 3) and the methods employed to measure pulse duration have limited precision at this scale.

**Table 5:** **Statistical comparisons between field and laboratory recordings for female sounds from *Ophidion rochei***

**Variables**	**Methods**	**Results**
**Pulse number**	M-W *U* test	N = 76, U = −1.27, p > 0.05
**Call duration**	M-W *U* test	N = 76, U = −1.07, p > 0.05
**Pulse period**	M-W *U* test	N = 354, U = 0.48, p > 0.05
**Pulse duration**	M-W *U* test	N = 431, U = −4.37, p < 0.05
**Fundamental**	M-W *U* test	N = 68, U = −0.37, p > 0.05
**1**^**st**^**harmonic**	M-W *U* test	N = 76, U = 77, p > 0.05
**2**^**nd**^**harmonic**	M-W *U* test	N = 77, U = −0.82, p < 0.05

M-W *U* test: *U* test of Mann-Whitney. Significance level is determined at p < 0.05.

#### Juvenile recordings

Juveniles were found to emit spontaneous sounds which waveforms were similar to female calls (Figure 6). However, the signal to noise ratio was not high enough in most of the files to permit a quantitative comparison with calls from adults. For a few of the loudest sounds it was possible to perform an analysis. Call duration (27.3 ± 15.6 ms, N = 4), pulse number (5.5 ± 1.7 ms, N = 4), pulse period (5.9 ± 2 ms, N = 18), and pulse duration (0.9 ± 0.5 ms, N = 22) were measured. Juvenile calls were found to have longer pulse period durations than female calls recorded in captivity (Mann–Whitney *U* test, p < 0.05). No differences were observed for the other three variables (p > 0.05). The sounds contained harmonics and fundamental frequency was situated between 162 Hz and 352 Hz. Additional data are required for a precise description of spectral characteristics.

## Discussion

In the present study, three morphotypes were described for *O*. *rochei* on the basis of sonic apparatus morphology. Sounds were recorded for each morphotype: juveniles, females, and males. The latter generally emit long calls that differ dramatically from female and juvenile sounds. It is the first time that female and juvenile sounds are described in Ophidiidae. These new data provide first evidence of the tight relationship between morphology of sonic apparatus and sound characteristics in this family.

### Sonic apparatus morphology

Casadevall investigated the relationship between gonadal maturation and size in *O*. *rochei*[53]. The smallest female that displayed mature gonads measured 136 mm in TL. When female total length exceeded 155 mm, gonads were always fully mature. In males, gonads reached complete maturation between 166 and 176 mm. The parallel between sonic apparatus ontogeny and gonadal maturation [53] strongly indicates that the complex male sonic apparatus morphology is a secondary sexual character that develops during puberty. The juvenile morphotype of *O*. *rochei* has the simplest sonic apparatus: three pairs of sonic muscles, neural rocker on the first vertebrae and slight modifications of the first and third epineurals. During gonadal maturation, the sonic apparatus undergoes weak transformation in females and extraordinary modifications in males that include differentiation of novel structures. X-ray photographs revealed the development of the ‘rocker bone’, the internal tube, the neck of the swimbladder, and the appearance of the gelatinous substance. Casadevall [46] did not find a rocker bone in individuals under 130 mm in TL. On X-ray photographs, the smallest fish with a rocker bone was 133 mm in TL, and was visible only as a small white dot. This fish also had a slight bump in the posterior part of the swimbladder, which indicates that the internal tube can develop earlier than suggested by the regression of Figure 4 (around 160 mm in TL). Males display also a more dramatic development of the first vertebrae (neural rocker) and associated bones (wing-like epineurals, swimbladder plate), the occipital region, and the sonic muscles. During adult stages no additional structures are observed but size-correlated variation in internal tube length indicates that this structure grows beyond sexual maturity. Observations of the sonic morphology of *O*. *rochei* are in agreement with previous studies on the ontogeny of sonic apparatus in fishes. In juveniles sonic muscles are absent [27] or monomorphic [27,29,31,32] and morphological differences between adult males and females appear related to sexual maturation [27,29,31,32]. Such changes are probably mediated by androgen hormones (see: [54]). In most fish taxa, however, sonic muscle changes are usually restricted to hypertrophy and ultrastructural modifications. However, in Ophidiiformes, the sexual dimorphism can also involve swimbladder shape and the ligaments and bony elements of the sonic apparatus [36,37,42,45,55]. *Ophidion rochei* represents an additional, and particularly extreme example of sexual dimorphism among Ophidiidae [36,37,42,45].

According to the parsimony principle, the fact that the rocker bone in males is a highly derived character and that the sonic apparatus morphotypes of female and juvenile *O*. *rochei* are similar and less complex, the sound producing apparatus of the male seems correspond to a case of peramorphosis. The ontogenetic trajectory of the male takes on hitherto unseen traits with the development of the rocker bone, the swimbladder and associated vertebral components.

With the exception of sonic muscles in some Sciaenidae species [27], the appearance of new structures of the sonic apparatus during sexual maturation had never been reported in fish. However, secondary sexual characters are common in vertebrates as external features. A very well-known example is the mane of lions which is only present in adult males [56]. Many examples have been documented in teleosts. In *Poecilia reticulata* (guppy), all juveniles display the same color pattern and shape [57,58]. In late juvenile stages, future males start to display conspicuous colors, a more developed tail and a gonopodium [57-59]. In nursery fish, *Kurtus gulliveri*, only adult males have a hook on the head that, after mating, holds gelatinous egg [60]. Note that secondary sexual characters of males are not always irreversible. The sexual dichromatism in fish can be permanent, seasonal, or ephemeral [61]. Seasonal variations in male morphology were also reported. For example, the head crest of male *Salaria pavo* increases in size during breeding season [62]. The nuptial tubercles of many male fish also disappear a few weeks after the breeding season [63]. In *O*. *rochei*, the sonic apparatus underwent very dramatic modifications (mineralized structures) observed during a complete year. Moreover, the internal tube size of adult males was correlated with fish total length. All evidence thus far indicates that the sexual dimorphism is permanent. However, an additional, more subtle effect of season on male sonic muscles cannot be excluded because a seasonal hypertrophy of sonic muscles was reported in the ophidiid *Lepophidium profondurum*[34].

### Sound production

Male sounds from both the field and captivity lasted a few seconds and showed an original acoustic signature in the pattern of pulse period [9]. This should allow female fish to consistently and easily identify male fish despite the abundance of physical and biological sounds in natural environments. Some differences, however, were noted between sounds in captivity and in the field. These differences could be attributed to environmental factors, such as seawater temperature, or intrinsic causes such as fish size [20-23]. A trend between pulse period and temperature is evident among existing datasets (14 to 21°C in captivity, 21.5°C in the field, and 23.5°C from Parmentier *et al*. [9]): shorter pulse periods were recorded at higher temperatures. Temperature-related variation in sound frequency spectra is less apparent because of the large variability observed in sounds recorded in captivity. High frequency sounds obtained in aquaria could be due to artifacts related to recording conditions in tanks (resonance). Despite the presence of these high pitched sounds, many male calls recorded in tanks had a frequency pattern very close to calls from the field (Figure 8). However, 1st and 2nd main frequencies described by Parmentier *et al*. [9] in September 2008 were 226 ± 1 Hz and 410 ± 1 Hz, which is slightly higher than data from July 2010 (191 ± 7 Hz, N = 19 and 355 ± 59 Hz, N = 19). This is in agreement with the tendency of fish sounds to increase in frequency with higher temperature [21].

The strongly dimorphic sound production anatomy of males and females corresponds to dramatic differences in sound waveforms and in frequency characteristics. Temporal and spectral characteristics from female sounds recorded in the laboratory were highly similar to sounds recorded from putative females in the field, with no discernible differences in call duration, pulse number, pulse period, fundamental frequency, and 1st harmonic as shown in table 5. These field recordings indicate that females may also produce nocturnal vocalizations.

Juveniles were recorded at water temperatures of 13-14°C and the period of their sounds was significantly longer than in females. Temperature may affect sound characteristics and partially explain differences between females and juveniles. Because of the small sample of juvenile sounds, however, interpretations of these data must be considered with caution. Though notably the very similar characteristics between most features of juvenile and female sounds, and the common dissimilarity of these sounds compared to male calls support the important relationship between morphology and sound characteristics.

### Implications for sound mechanisms

In many respects, the sonic apparatus of male *O*. *rochei* is more different from the sonic apparatus of female *O*. *rochei* than it is from males of many other *Ophidion* species. The male sound production apparatus shows more morphological modifications that may reflect a greater specialization for sound production. Compared with females, males show more development of the occipital region and neural rocker for the insertion of muscles powerful enough to move heavy rocker bone back and forth [47] and to counterbalance the suction engendered in the swimbladder. The constraint posed by rapid pressure changes may explain the development of thick elastic layers into the internal tube acting as a pressure-release valve at the back of the swimbladder [9]. Females lack a rocker bone: less developed muscles insert on smaller surfaces and the swimbladder lacks a pressure-release valve. As a whole, it is easier to vibrate the swimbladder in females. Thus, inertial considerations derived from morphological data, together with sound characteristics, indicate substantial differences in the sound production mechanism of male and female.

Mainly because of the long pulse period of their sounds, Ophidiiformes (carapids and ophidiids) are thought to use slow sonic muscles [9,44]. In *Carapus acus*, the sonic muscle was shown to tetanize in the vicinity of 10 Hz [44]. However, the pulse period, presence of harmonics, and shape of the oscillogram of sounds produced by female *O*. *rochei* are characteristics that are typical of fish sounds produced by high-speed muscles [21,64-68]. These sound data suggest that the call fundamental frequency corresponds to the contraction rate of sonic muscle. In female *O*. *rochei*, fundamental frequency was around 250 Hz, which would correspond to a very fast sonic muscle contraction rate. In *Prionotus carolinus* (Triglidae), bilateral alternation in the contraction of paired muscles allows each muscle to contract at half the fundamental frequency of a voluntarily calling fish [69]. Based on sonic characteristics, the muscle physiology between males and females is expected to be vastly different. However, despite the relatively modest pulse rate of *Cynoscion regalis* sounds (*ca*. 20 Hz), Connaughton *et al*. [70] suggested that each pulse results from a very fast contraction of sonic muscles. Thus, it is possible that male *O*. *rochei* are capable of fast contractions but this ability cannot be deduced from sound production. Electrophysiological data are definitely needed to test this hypothesis.

### Evolutionary concerns

The sonic apparatus of male *O*. *rochei* undergoes substantial modification during ontogeny, resulting in dramatic sexual dimorphism. Sexual dimorphism and dichromatism are common in vertebrates (e.g., [61,71-73]) and secondary sexual characters can play a role in sex recognition, mate choice, or both. For example, male colors are involved in female mate choice in several fish clades [61]. In *Poecilia reticulata*, morphological traits, such as tail size, were also demonstrated to influence female choice [59].

In mammals [71], secondary sexual characters are generally more pronounced in diurnal species. Dimorphism in characters, such as canine teeth, is more important in diurnal than nocturnal species because these characters are more easily detected in daylight [71], while dark environments restrict the efficiency of visual communication for species and sex recognition. As a result, many marine species have evolved bioluminescent organs (photophores) [74]. In fish, photophore distribution has species-specific patterns that are often sexually dimorphic [74]. Many fish clades acquired alternative communication cues (electric, acoustic, and chemical signals) [75] effective even in dark environments.

Like many Ophidiidae [35], *O*. *rochei* is mainly active in the dark, yet has no photophores and displays no striking external dimorphism. Conspicuous color dichromatism is also absent in both sexes. Thus external features are expected to have little effect on mate choice and sex recognition. Sound characteristics in this species, however, could be used for species and sex recognition. The call of male *O*. *rochei* is conspicuous because of its long duration and the unique pattern observed in its pulse period. Long signals with highly repetitive sounds are more likely detected. They also favor the localization of the sound source because targeted fish may evaluate variation in sound intensity as they are moving. Pulse period could be a key for species recognition because several studies suggest that temporal characteristics of sounds are more informative and reliable for fish communication than spectral features [76,77]. Moreover, pulse period seems to be the less distorted sound characteristic during propagation in shallow water [78]. Hence species-specific patterns of male calls should be preserved during sound propagation in comparison to other characteristics like frequency and pulse duration.

For these reasons, the acoustic sexual dimorphism of *O*. *rochei* may be of major importance for sex recognition and mate localization in the dark active environment of this species. Behaviors associated with sound production of this fish remain unknown. Since male calls in *Ophidion marginatum* are related to reproduction [8], we hypothesize that acoustic communication favors the reproductive success of *O*. *rochei*. Variations in secondary sexual characters [79] and communication cues [80] are thought to promote speciation and thus it is likely that acoustic communication is involved in the evolutionary success and the important radiation of the family.

## Conclusions

Males of *O*. *rochei* are able to produce sounds that differ greatly from female and juvenile calls. This dichotomy in sound characteristics is related to major differences in sonic apparatus morphology. During male sexual maturation, new structures (rocker bone, internal tube, swimbladder plate) involved in the sonic apparatus appear and develop continuously. This peramorphosis corresponds to the development of secondary sexual characters that have to be advantageous in species living in a dark environment.

## Methods

### Fish collection

Twenty-four Fish, 67 to 217 mm TL (total length), were caught during May and July 2010 near the Cetina estuary in Dùlce-Glàva, Croatia (43°26 N, 16°40 E). They were trapped with a beach seine (22 m long, mesh size of 4 mm at the outer wing and 2 mm at the central part) in shallow water (<2 m depth) from 21:00 to 02:00. Then, they were held for one week in a 250 l tank at the Institute of Oceanography and Fisheries in Split (Croatia). Finally, they were transported to Liège (Belgium) and kept in a 1000 l tank with a 0.1 m high sandy bottom. In August 2010, an additional specimen was caught with a small hand net during a scuba dive at Banyuls-sur-mer, France (42°28 N, 3°08 E).

Eighty-nine additional swimbladders from *O*. *rochei* sampled between 1986 and 1988 along the Costa Brava (Spain) were utilized. Specimens were measured, dissected, and sexed by Casadevall [53] and their swimbladder were fixed in formaldehyde (7%) and kept in ethyl-alcohol (70%).

Casadevall [53] studied the gonads of 223 specimens of *Ophidion rochei*. These gonads were never mature before individuals reach a total length of 136 mm in females and 166 mm in males. Specimens with a TL under these thresholds were thus considered immature. However, traces of the future rocker bone were already observed in males just over 130 mm in TL [53]. Consequently, the samples were divided in 3 classes: juveniles (<130 mm in TL), females (>130 mm in TL and no rocker bone), immature and mature males (>130 mm in TL and first evidence or presence of a rocker bone).

### Gross morphology of the sonic apparatus

Twelve individuals were euthanized with an overdose of MS 222, fixed in formaldehyde (7%) during 15 days and transferred in norvanol. Three females (154 mm, 175 mm and 187 mm in TL) and two juveniles (77 mm and 98 mm in TL) were stained with Alizarin Red to visualize osseous structures [81]. The other individuals were carefully dissected to study the swimbladder, the ligaments, and the muscles of the sound production apparatus. The general morphology of the sonic apparatus of females and juveniles was examined with a binocular microscope (Leica, Wild M10) coupled to a camera lucida and compared with previously described males [9].

The anterior part of swimbladders from Costa Brava had previously been removed for studies on the ‘rocker bone’. However, the posterior part remained well preserved and allowed the measurement of the internal tube [see: [46]] present in male swimbladders.

Living fish collected from Croatia were X-ray photographed in July 2010 (adults, N = 14 and juveniles, N = 10) and the eleven surviving fish were X-rayed again in May 2011 (adults, N = 10 and juvenile, N = 1). The fish collected from France was X-ray photographed regularly (±each month) from August 2010 to September 2011. X-ray photographs were performed at the Veterinary Institute of the University of Liège with a DigiVeX FP (MEDEX Loncin S.A., Belgium) under 43 kV and 10 mAs^-1^. All fish were anesthetized with MS 222 (150 mg/l) to prevent them for moving during the X-ray sessions. The skeleton, the swimbladder, and the position and size of the rocker bone (in morphotypes where present) were observed on X-ray photographs. All experimental procedures were approved by the University of Liège Institutional Animal Care and Use Committee.

### Sound recordings

#### Male recordings

Two immature males (133 and 163 mm TL) and four mature males (168, 169, 191, and 206 mm TL) were placed with three females in a rectangular plastic tank of 900 l. From January to April 2011, seawater temperature was kept at 14°C and the light period was lengthened gradually (from 8:00 to 10:45 h of daylight) to mimic winter conditions in Adriatic Sea. Temperature was increased from 14°C in April to 19.5°C in June and light periods were lengthened from 10:45 to 15:00 h to simulate the spring. Because these calls are thought to play a role in female attraction, nets were placed into the tank from May to June to separate males from females. Sounds were recorded with a Digital Spectrogram Long-Term Acoustic Recorder (DSG, Loggerhead Instruments, Sarasota, FL, USA). This apparatus is composed of a hydrophone (186 dB re 1 V/μPa) coupled to a digital acquisition board. It recorded ten minutes each half hour during night time. Recordings investigated in this study were all performed at a sample rate of 20,000 Hz.

#### Female recordings

Four females (198, 200, 205, and 209 mm TL) were free to swim in a tank of 700 l. Sounds were recorded with the DSG from 21 September 2010 to 19 October 2010. Only females were present in the tank during recording periods. The DSG hydrophone recorded periods of ten minutes each half hour from 19:00 to 07:00, UTC + 1. During the recording period (21 September to 19 October) water temperature varied between 18°C and 25°C.

#### Juvenile recordings

Three juveniles (67, 78, and 87 mm TL) were recorded in a 250 l tank in Croatia. Temperature averaged 13-14°C. The same hydrophone and recording conditions previously described were used.

#### Field recordings

In July 2010, the DSG was used mainly during night time (21:30 to 04:30, UTC + 1) and recorded 10 min each 30 min. It was placed 70 m from the beach of Dùlce-Glàva at a depth of 2 m. These recordings were investigated for sounds produced by *O*. *rochei*.

### Sound analyses

Sounds were digitized at 20 kHz (16 bit resolution) and analyzed using Avisoft SAS-Lab Pro 4.5. Based on Akamatsu *et al*. [82], the minimum resonant frequency calculated for recording tanks from Liège was greater than 1.7 kHz. The minimum resonant frequency was higher in the tank from Croatia. To avoid resonant effects and reduce low frequency background noise, a band pass filter that kept frequencies between 0.05 and 1.7 kHz was applied prior to each analysis. This step was achieved thanks to the FIR (Finite Impulse Response) band pass filter of Avisoft software.

Spectral analyses were performed in Avisoft with the power spectrum logarithmic function (Hamming window). The power spectrum graphs obtained displayed the distribution of sound energy across frequencies. Harmonic sounds are indicated by the presence of several regular spaced peaks on the power spectrum, in which the harmonic peaks are multiples of the lowest peak (fundamental frequency). Non harmonic sounds are dominated by one or several frequencies with no conserved mathematical relationship between them. The highest frequency peak or peaks of non-harmonic sounds were categorized as main frequencies. Because of the relatively long duration of male calls, the results of the FFT function were averaged over 41 points to clarify graphical illustration.

A semi-automated method with Avisoft software was used to measure call duration, number of pulses in a call, pulse period duration, and pulse duration. Sounds were defined as a series of one or more pulses (Figure 6). Inter-pulse interval (data not shown in the results) is the duration of time between a pulse offset and the onset of the next pulse (Figure 6). Pulse period is the duration of time between successive pulse onsets. Thus, pulse period is the repeated unit that constitutes the multiple-pulsed sounds. All analyses were performed on the root mean squared signal (function ‘RMS with exponential moving average + decimation’ in Avisoft software) with the peak search with hysteresis function (15 dB in males with start/stop threshold at -15 dB and 1 dB in females and juveniles with a start/stop threshold at -1 dB). The hysteresis was lower for female and juvenile sounds because the pulse amplitude to inter-pulse amplitude ratio was lower, which could affect pulse duration accuracy slightly, but increased the accuracy of call duration, pulse period, and pulse number provided by the program. The original waveform was transformed in RMS signal because it allowed a better detection of peak boundaries: the onsets and offsets of pulses were sharper and thus easier to detect and define. The application of thresholds based on the mean RMS of background noise highly reduced the number of missed detections. In a few cases, peaks from background noise were automatically selected and needed to be removed manually. However, pulse and pulse period durations displayed by the program were never modified manually to avoid subjective modifications. For male sounds, additional variables were deducted from analysis output. As described in Parmentier *et al*. [9], pulse period in male sounds shows a cyclic variation. Its duration rises before alternating between long and short pulse period durations. ‘Long pulse period’ and ‘short pulse period’ were considered as two additional variables. The variable ‘alternation start’ corresponded to the pulse number when the pulse period alternation pattern was initiated. This variable was objectively defined as the first pulse period that 1) decreased by 5 ms from the preceding pulse period, and 2) was followed by a series of alternating long and short pulse periods that continued until the end of the sound. Thus ‘alternation start’ refers to the number of the pulse just after the first shortened pulse period. For a graphical illustration of alternation in male call *O*. *rochei* see Parmentier *et al*. [9].

Statistical analyses were performed with STATISTICA 9.1 and Graphpad Prism 5 (Graphpad Software, Inc.). The latter was also employed for graphical illustration. In addition to elementary statistics, the normality of data was tested with Kolmogorov-Smirnov and Shapiro-Wilk tests to determine whether parametric tests were appropriate. Variables that did not violate assumptions of normality were compared with a Student’s *t*-test, while variables with a non-normal distribution were tested with a Mann-Whitney *U* test.

## Competing interests

The authors declare that they have no competing interests.

## Authors’ contributions

KL sampled fish, made analyses, and wrote the manuscript. BK revised the content and form of the manuscript. DB and DJ took part to field campaigns. CM supplied additional samples. PE took part to field campaigns, revised manuscript, and gave final approval. All authors read and approved the final manuscript.

## Authors’ information

KL is a PhD student of the FRIA at the University of Liège and he works on acoustic communication in Ophidiiform. BK is a post-doctoral researcher at the University of Liège and he works on acoustic and electrical communication in catfish. DJ is professor at the University of Split and Scientific Advisor at the Institute of oceanography and fisheries in Split. DB is a PhD student and also the assistant of DJ at the Institute of oceanography and fisheries in Split. CM is professor at the University of Girona (Spain). She did her PhD on some anguilliform fish from Mediterranean Sea in which *Ophidion rochei*. EP is the head of the Laboratory of functional and evolutionary morphology at the University of Liège and the advisor of KL.

## References

[B1] Bass A McKibben J Neural mechanisms and behaviours for acoustic communication in teleost fish Prog Neurobiol 2003 69 1 26 10.1016/S0301-0082(03)00004-2 12637170

[B2] Courtenay WR McKittrick FA Sound-producing mechanisms in carapid fishes, with notes on phylogenetic implications Mar Biol 1970 7 131 137 10.1007/BF00354916

[B3] Mann D Lobel P Acoustic behaviour of the damselfish *Dascyllus albisella*: behavioural and geographic variation Environ Biol Fishes 1998 51 421 428 10.1023/A:1007410429942

[B4] Moulton J The acoustical behaviour of some fishes in the Bimini (Bahama Island) area Marine Biol Lab 1958 114 357 375 10.2307/1538991

[B5] Takemura A Acoustical behavior of the freshwater goby *Odontobutis obscura* Bull Jpn Soc Sci Fisheries 1984 50 561 564 10.2331/suisan.50.561

[B6] Myrberg AA Tavolga WN, Popper AN, Fay R Sound communication and interception of fishes Hearing and sound communication in fishes 1981 New-York: Springer 395 452

[B7] Parmentier E Vandewalle P Lagardère J-P Sound-producing mechanisms and recordings in *Carapini* species (Teleostei, Pisces) J Comp Physiol A 2003 189 283 292 10.1007/s00359-003-0401-7 12743733

[B8] Mann DA Bowers-Altman J Rountree RA Sounds produced by the striped cusk-eel *Ophidion marginatum* (Ophidiidae) during courtship and spawning Copeia 1997 3 610 612

[B9] Parmentier E Bouillac G Dragicevic B Dulcic J Fine ML Call properties and morphology of the sound-producing organ in *Ophidion rochei* (Ophidiidae) J Exp Biol 2010 213 3230 3236 10.1242/jeb.044701 20802126

[B10] Parmentier E Boyle KS Berten L Brié C Lecchini D Sound production and mechanism in *Heniochus chrysostomus* (Chaetodontidae) J Exp Biol 2011 214 2702 2708 10.1242/jeb.056903 21795566

[B11] Tricas T Kajiura SM Kosaki RK Acoustic communication in territorial butterflyfish: test of the sound production hypothesis J Exp Biol 2006 209 4994 5004 10.1242/jeb.02609 17142688

[B12] Nakazato M Takemura A Acoustical behavior of the japanese parrot fish *Oplegnathus fasciatus* Nippon Suisan Gakkaishi 1987 53 967 973 10.2331/suisan.53.967

[B13] Miyagawa M Takemura A Acoustical behavior of scorpaenoid fish *Sebasticus marmoratus* Bull Jpn Soc Sci Fisheries 1986 52 411 415 10.2331/suisan.52.411

[B14] Slabbekoorn H Bouton N van Opzeeland I Coers A ten Cate C Popper AN A noisy spring: the impact of globally rising underwater sound levels on fish Trends in Ecol Evol 2010 25 419 427 10.1016/j.tree.2010.04.005 20483503

[B15] Ladich F Fine ML Ladich F, Collin SP, Moller P, Kapoor BG Sound-generating mechanisms in fishes: a unique diversty in vertebrates Communication in Fishes. Volume 1 2006 Enfield (NH), USA: Science Publishers 3 34

[B16] Parmentier E Diogo R Ladich F, Collin SP, Moller P, Kapoor BG Evolutionary trends of swimbladder sound mechanisms in some teleost fishes Communication in Fishes. Volume 1 2006 Enfield (NH), USA: Science Publisher 45 70

[B17] Bradbury JW Vehrencamp SL Bradbury JW, Vehrencamp SL Chapitre 4: sound production Principles of animal communication. Volume 1 1998 Canada: Sinauer Associates Inc

[B18] Demski LS Gerald JW Popper AN Central and peripheral mechanisms of teleost sound production Am Zool 1973 13 1141 1167

[B19] Feher JJ Waybright TD Fine ML Comparison of sarcoplasmic reticulum capabilities in toadfish (*Opsanus tau*) sonic muscle and rat fast twitch muscle J Muscle Res Cell Motil 1998 19 661 674 10.1023/A:1005333215172 9742450

[B20] Connaughton MA Fine ML Taylor MH Review. Weakfish sonic muscle: influence of size, temperature and season J Exp Biol 2002 205 2183 2188 12110652 10.1242/jeb.205.15.2183

[B21] Fine ML Seasonal and geographical variation of the mating call of the oyster toadfish *Opsanus tau* L Oecologia 1978 36 45 57 10.1007/BF00344570 28309226

[B22] Spargue MW Luczkovich JJ Do striped cusk-eels *Ophidion marginatum* (Ophidiidae) produce the “chatter” sound attribuated to weakfish *Cynoscion regalis* (Scianidae)? Copeia 2001 3 854 859

[B23] Papes S Ladich F Effects of temperature on sound production and auditory abilities in the striped raphael catfish *Platydoras armatulus* (Family Doradidae) PLoS One 2011 6 1 10 10.1371/journal.pone.0026479 PMC3195728 22022618

[B24] Marsh RL Ontogenesis of contractile properties of skeletal muscle and sprint performance in the lizard *Dipsosaurus dorsalis* J Exp Biol 1988 137 119 139 3209964 10.1242/jeb.137.1.119

[B25] Archer SD Atlringham JD Johnston IA Scaling effects on the neuromuscular system, twitch kinetics and morphometrics of the cod, *Gadus morhua* Marine Behav Physiol 1990 7 137 146

[B26] Bennett AF Garland TJ Else PL Individual correlation of morphology, muscle mechanics and locomotion in a salamander Am J Physiol 1989 256 1200 1208 10.1152/ajpregu.1989.256.6.R1200 2735445

[B27] Hill GL Fine ML Musick JA Ontogeny of the sexually dimorphic sonic muscle in three sciaenid species Copeia 1987 3 708 713

[B28] Ramcharitar JU Gannon DP Popper AN Bioacoustics of fishes of the family Scianidae (croackers and drums) Trans Am Fish Soc 2006 135 1409 1431 10.1577/T05-207.1

[B29] Fine ML Burns NM Harris TM Ontogeny and sexual dimorphism of sonic muscle in the oyster toadfish Canadian J Zool 1990 68 1374 1381 10.1139/z90-205

[B30] Brantley RK Bass A Alternative male spawning tactics and acoustic signals in the plainfin midshipman fish, *Porichthys notatus* Girard (Teleostei, Batrachoididae) Ethology 1994 96 213 232

[B31] Brantley RK Tseng J Bass AH The ontogeny of inter- and intrasexual vocal muscle dimorphisms in a sound producing fish Brain, Behav Evol 1993 42 336 349 10.1159/000114170 8275300

[B32] Loesser KE Rafi J Fine ML Embryonic, juvenile, and adult development of the toadfish sonic muscle Anat Rec 1997 249 469 477 10.1002/(SICI)1097-0185(199712)249:4<469::AID-AR6>3.0.CO;2-M 9415454

[B33] Connaughton MA The effects of seasonal hypertrophy and atrophy on fiber morphology, metabolic substrate concentration and sound characteristics of the weakfish sonic muscle J Exp Biol 1997 200 2449 2457 9343856 10.1242/jeb.200.18.2449

[B34] Nguyen TK Hsung L Parmentier E Fine ML Seasonal variation in sonic muscles in the fawn cusk-eel *Lepophidium profundorum* Biol Lett 2008 4 707 710 10.1098/rsbl.2008.0383 18812307 PMC2614160

[B35] Nielsen J Cohen D Markle D Robins C Ophidiiform fishes of the world (order Ophidiiformes) 1999 Rome: FAO

[B36] Carter JH Musick JA Sexual dimorphism in the deep-sea fish *Barathrodemus manatinus* (Ophidiidae) Copeia 1985 1 69 73

[B37] Courtenay WR Sexual dimorphism of the sound producing mechanism of the striped cusk eel, *Rissola marginata* (Pisces: Ophidiidae) Copeia 1971 2 259 268

[B38] Campagna AJ Comparative morphology and phylogenetic significance of sound-producing mechanisms in some Brotulidae (Osteichthys, Ophidioidea) 1973 USA: Boston University

[B39] Howes GJ Notes on the anatomy and classification of Ophidiiform fishes with particular reference to the abyssal genus *Acanthonus* Günther, 1878 Bull Bristish Museum (Nat History) 1992 58 95 131

[B40] Lagardère J-P Millot S Parmentier E Aspects of sound communication in the pearlfish *Carapus boraborensis* and *Carapus homei* (Carapidae) J Exp Zool 2005 303A 1066 1074 10.1002/jez.a.230 16254913

[B41] Parmentier E Fine ML Vandewalle P Ducamp J-J Lagardère J-P Sound production in two carapids (*Carapus acus* and *C. mourlani*) and through the sea cucumber tegument Acta Zool 2006 87 113 119 10.1111/j.1463-6395.2006.00221.x

[B42] Rose JA Anatomy and sexual dimorphism of the swim bladder and vertebral column in *Ophidion holbrooki* (Pisces: Ophidiidae) Bull Mar Sci 1961 11 280 308

[B43] Parmentier E Fontenelle N Fine ML Vandewalle P Henrist C Functional morphology of the sonic apparatus in *Ophidion barbatum*(Teleostei, Ophidiidae) J Morphol 2006 267 1461 1468 10.1002/jmor.10496 17103392

[B44] Parmentier E Lagardère J-P Braquegnier J-B Vandewalle P Fine ML Sound production mechanism in carapid fish: first example with a slow sonic muscle J Exp Biol 2006 209 2952 2960 10.1242/jeb.02350 16857879

[B45] Fine ML Hsung L Nguyen BB Rountree RA Cameron TM Parmentier E Functional morphology of the sonic apparatus in the fawn cusk-eel *Lepophidium profundorum* (Gill, 1863) J Morphol 2007 268 953 966 10.1002/jmor.10551 17674354

[B46] Casadevall M Matallanas J Carrasson M Muñoz M Morphometric, meristic and anatomical differences between *Ophidion barbatum* L., 1758 and *O. rochei* Müller, 1845 (Pisces, Ophidiidae) Publicaciones Especiales - Instituto Español de Oceanografìa 1996 21 45 61

[B47] Parmentier E Compère P Casadevall M Fontenelle N Cloots R Henrist C The rocker bone: a new kind of mineralised tissue? Cell Tissue Res 2008 334 67 79 10.1007/s00441-008-0665-x 18665393

[B48] Markle D Olney J Systematics of the pearlfishes (Pisces, Carapidae) Bull Mar Sci 1990 47 269 410

[B49] Matallanas J Casadevall M Present day distribution and historical biogeography of the tribe Ophidiini (Ophidiiformes, Ophidiidae, Ophidiinae) from East Tropical Atlantic (CLOFETA area) and the North-East Atlantic and Mediterranean (CLOFNAM area) Cah Biol Mar 1999 40 135 140

[B50] Dulcic J Fisrt record of juvenile cusk-eel *Ophidion rochei* (Ophidiidae) in the Adriatic Cybium 2001 25 289 290

[B51] Fischer W Bauchot ML Schneider M Fiches FAO d’identification des espèces pour les besoins de la pêche 1987 Rome: Méditerranée et Mer Noire

[B52] Jardas I Jadranska ihtiofauna 1996 Zagreb: Skolska knjiga (in Croatian) 552 p

[B53] Casadevall M Aspectes anatòmics i biològics d’alguns anguilliformes i ophidiiformes del mediterrani occidental 1991 PhD thesis. Universidad autònoma de Barcelona, Departament de Biologia Animal, Biologia Vegetal i Ecologia

[B54] Fine ML Endocrinology of sound production in fishes Mar Freshw Behav Physiol 1997 29 23 45 10.1080/10236249709378999

[B55] Parmentier E Vandewalle P Further insight on carapid-holothuroid relationships Mar Biol 2005 146 455 465 10.1007/s00227-004-1467-7

[B56] Gould JL Gould CG Sexual selection: mate choice and courtship in nature 1989 2 New-York, USA: Scientific American Library

[B57] Houde AE Sex, color, and mate choice in guppies 1997 New Jersey: Princeton University Press

[B58] Magellan K Magurran AE The effect of social environment during ontogeny on life history expression in the guppy *Poecilia reticulata* J Fish Biol 2009 74 2329 2337 10.1111/j.1095-8649.2009.02245.x 20735556

[B59] Bischoff RJ Gould JL Rubenstein DI Tail size and female choice in the guppy (*Poecilia reticulata*) Behav Ecol Socioecol 1985 17 253 255 10.1007/BF00300143

[B60] Berra TM Humphrey JD Gross anatomy and histology of the hook and skin of forehead brooding male nurseryfish, *Kurtus gulliveri*, from northern Australia Env Biol Fishes 2002 65 263 270 10.1023/A:1020523905635

[B61] Kodric-Brown A Sexual dichromatism and temporary color changes in the reproduction of fishes Am Zool 1998 38 70 81

[B62] Fagundes T Gonçalves DM Oliveira RF Female mate choice and mate search tactics in a sex role reversed population of peacock blenny *Salaria pavo* J Fish Biol 2007 71 77 89 10.1111/j.1095-8649.2007.01466.x

[B63] Wiley ML Colette BB Breeding tubercles and contact organs in fishes: their occurence, structure, and significance Bull Am Mus Nat Hist 1970 143 143 216

[B64] Tavolga WN Tavolga WN Sonic characteristics and mechanisms in marine fishes Marine bio-acoustics 1964 New-York: Pergamon Press 195 211

[B65] Cohen MJ Winn HE Electrophysiological observations on hearing and sound production in the fish, *Porichthys notatus* J Exp Biol 1967 165 355 370 10.1002/jez.1401650305 6076901

[B66] Skoglund CR Functional analysis of swimbladder muscles engaged in sound production of the toadfish J Biophys Biochem Cytology 1961 10 187 200 10.1083/jcb.10.4.187 PMC2225107 19866593

[B67] Millot S Vandewalle P Parmentier E Sound production in red-bellied piranhas (*Pygocentrus nattereri*, Kner): an acoustical, behavioural and morphofunctional study J Exp Biol 2011 214 3613 3618 10.1242/jeb.061218 21993790

[B68] Fine ML Movement and sound generation by the toadfish swimbladder J Comp Physiol A 2001 187 371 379 10.1007/s003590100209 11529481

[B69] Connaughton MA Sound generation in searobin (*Prionotus carolinus*), a fish with alternate sonic muscle contraction J Exp Biol 2004 207 1643 1654 10.1242/jeb.00928 15073197

[B70] Connaughton MA Taylor MH Fine ML Effects of fish size and temperature on weakfish disturbance calls: implications for the mechanism of sound generation J Exp Biol 2000 203 1503 1512 10751166 10.1242/jeb.203.9.1503

[B71] McPherson FJ Chenoweth PJ Mammalian sexual dimorphism Anim Reprod Sci 2012 131 109 122 10.1016/j.anireprosci.2012.02.007 22482798

[B72] Björklund M Coming of age in fringillid birds: heterochrony in the ontogeny of secondary sexual characters J Evol Biol 1991 4 83 92 10.1046/j.1420-9101.1991.4010083.x

[B73] Owens IP Short RV Hormonal basis of sexual dimorphism in birds: implications for new theories of sexual selection Trends Ecol Evol 1995 10 44 47 10.1016/S0169-5347(00)88967-3 21236951

[B74] Herring PJ Sex with the lights on? A review of bioluminescent sexual dimorphism in the sea J Mar Biol Assoc UK 2007 87 829 842 10.1017/S0025315407056433

[B75] Ladich F Colin SP Moller P Kapoor BG Communication in fishes: volume 1 and 2 2006 1 Enfield (NH), USA: Science Publishers

[B76] Wysocki LE Ladich F, Collin S, Moller P, Kapoor B Detection of communication sounds Communication in Fishes. Volume 1 2006 1 Enfied U.S.A: Science publishers

[B77] Winn HE Tavolga WN The biological significance in fish sounds Marine bio-acoustics 1964 New-York: Pergamon 213 231

[B78] Mann D Lobel P Propagation of damselfish (Pomacentridae) courtship sounds J Acoust Soc Am 1997 101 3783 3791 10.1121/1.418425

[B79] Higashi M Takimoto G Yamamura N Sympatric speciation by sexual selection Nature 1999 402 523 526 10.1038/990087 10591210

[B80] Streelman JT Danley PD The stages of vertebrate evolutionary radiation Trends Ecol Evol 2003 18 126 131 10.1016/S0169-5347(02)00036-8

[B81] Taylor W Van Dyke G Revised procedure for staining and clearing small fishes and other vertebrates for bone and cartilage study Cybium 1985 2 107 119

[B82] Akamatsu T Okumura T Novarini N Yan HY Empirical refinements applicable to the recording of fish sounds in small tanks J Acoust Soc Am 2002 112 3073 3082 10.1121/1.1515799 12509030

